# A nomogram incorporating red blood cell indices to predict post-stroke cognitive impairment in the intracerebral hemorrhage population

**DOI:** 10.3389/fnagi.2022.985386

**Published:** 2022-09-15

**Authors:** Yongzhe Gu, Fang Wang, Li Gong, Min Fang, Xueyuan Liu

**Affiliations:** ^1^Department of Neurology, Shanghai Tenth People’s Hospital, School of Medicine, Tongji University, Shanghai, China; ^2^Department of Neurology, The Second People’s Hospital of Yibin, West China Yibin Hospital, Sichuan University, Yibin, China

**Keywords:** intracerebral hemorrhage, cognitive impairment, risk factor, red blood cell indices, nomogram

## Abstract

**Background:**

Post-stroke cognitive impairment (PSCI) plagues 20–80% of stroke survivors worldwide. There is a lack of an easy and effective scoring tool to predict the risk of PSCI in intracerebral hemorrhage (ICH) patients. We aimed to develop a risk prediction model incorporating red blood cell (RBC) indices to identify ICH populations at risk of PSCI.

**Methods:**

Patients diagnosed with ICH at the stroke center were consecutively enrolled in the study as part of the development cohort from July 2017 to December 2018, and of the validation cohort from July 2019 to February 2020. Univariable and multivariable analyses were applied in the development cohort to screen the patients for PSCI risk factors. Then, a nomogram based on RBC indices and other risk factors was developed and validated to evaluate its performance in predicting PSCI occurrence.

**Results:**

A total of 123 patients were enrolled in the development cohort, of which 69 (56.1%) were identified as PSCI, while 38 (63.3%) of 60 patients in the validation cohort were identified as PSCI. According to the multivariate analysis, seven independent risk factors, including three RBC indices (hemoglobin, mean corpuscular volume, RBC distribution width), as well as age, education level, hematoma volume, and dominant-hemisphere hemorrhage were incorporated into the model. The nomogram incorporating RBC indices displayed good discrimination and calibration. The area under the receiver operating characteristic curve was 0.940 for the development cohort and 0.914 for the validation cohort. Decision curve analysis and clinical impact curve showed that the nomogram was clinically useful.

**Conclusion:**

RBC indices are independent and important predictors of PSCI. A nomogram incorporating RBC indices can be used as a reasonable and reliable graphic tool to help clinicians identify high cognition impairment-risk patients and adjust individualized therapy.

## Introduction

Post-stroke cognitive impairment (PSCI) refers to a series of syndromes from mild cognitive impairment to dementia after stroke and is one of the major complications caused by stroke (Zhang and Bi, 2020). It plagues 20–80% of stroke survivors worldwide and is a prevalent public health burden causing tremendous loss of social resources (Sun et al., 2014; Zhou et al., 2021). Intracerebral hemorrhage (ICH) is a subtype of severe hemorrhagic stroke (Lv et al., 2021). ICH accounts for 10–15% of all strokes, but 30–50% of stroke-related mortality, disability, and cost (Keep et al., 2012). Especially, cognitive impairment accompanied by progressive and irreversible clinical progression has been shown to be prevalent in high-risk ICH patients (Pasi et al., 2021). Information processing speed, executive function, memory, language, and visuo-spatial abilities are the most frequently identified cognitive domains affected by ICH (Zheng et al., 2019). Therefore, improving the prediction of PSCI in ICH patients is valuable.

International guidelines have recommended routine cognitive assessment for all stroke survivors due to their susceptibility to and characteristics of PSCI (Hachinski et al., 2006; Pendlebury and Rothwell, 2009). Early recognition of PSCI facilitates prompt intervention to avoid worse outcomes, recently attracting increased attention. Several studies have explored the PSCI prediction models. Dong et al. (2021) built a clinical model (DREAM-LDL) for PSCI. DREAM-LDL [Diabetes (fasting blood glucose level), Rating (NIHSS), level of Education, Age, baseline MoCA, and LDL-C level] had good predictive power for PSCI. Another PSCI model based on magnetic resonance spectroscopy imaging, which detects related indices of the bilateral prefrontal lobe, thalamus, basal ganglia, hippocampus, precuneus, and angular gyrus, also assists clinicians in estimating the risk of cognitive impairment after stroke (Yuan et al., 2021). Additionally, the model built with the severity of intracranial atherosclerotic stenosis as one of the predictors can be applied to evaluate cognitive impairment after minor ischemic stroke (Gong et al., 2021).

However, because of the time-consuming, complex, and expensive characteristics, these prediction models are relatively challenging to be popularized in clinical settings. Notably, studies on PSCI have primarily focused on ischemic stroke, and relatively few researchers have addressed risk factors associated with hemorrhagic PSCI in recent years. While ischemic stroke and hemorrhagic stroke are two subtypes with different pathogenesis, it is necessary to explore predictors for ICH patients with PSCI. Based on the above considerations, a cheap, fast, and simple hemorrhagic PSCI model is more ideal to be widely used as a prognostic guide in clinical practice.

A routine complete blood count is the most commonly measured laboratory test in hospital, for the advantage of being widely available, non-invasive, easy, economical, and rapid to perform (Gilev et al., 2017). Several studies have shown the significance of red blood cell (RBC) indices in predicting mortality, clinical outcomes, and functional rehabilitation after stroke (Roh et al., 2019; Pinho et al., 2021). In addition, increasing evidence suggests that RBC indices may also be indicative of cognitive impairment (Dlugaj et al., 2016). However, the association between RBC indices and hemorrhagic PSCI has not been explored much. Therefore, in this study, we aimed to investigate the association between RBC indices and PSCI risk among ICH patients, and further construct a nomogram incorporating RBC indices to identify high PSCI risk ICH populations.

## Materials and methods

### Study design and participants

**FIGURE 1:**
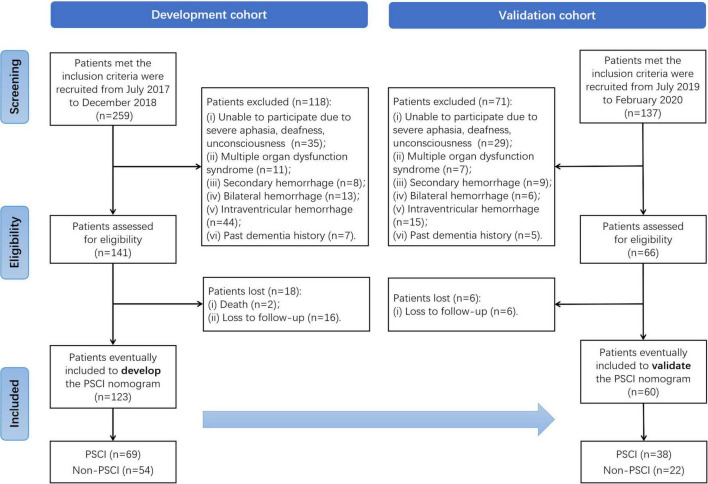
Flowchart of selection process.

We conducted a longitudinal and two-stage study to develop and then validate a PSCI prediction model in an ICH population. In the first stage, patients diagnosed with ICH by computed tomography (CT) were enrolled as part of the development cohort from July 2017 to December 2018 at the stroke center of Shanghai Tenth People’s Hospital. In the next stage, additional ICH patients were recruited as part of the validation cohort from July 2019 to February 2020. All patients recruited at the stroke center did not have hematological or RBC disease. Details of study design and patient selection are illustrated in a flowchart (Figure 1). The study was carried out in accordance with the Helsinki Declaration and was approved by the Ethics Committee of Shanghai Tenth People’s Hospital (SHSY-IEC-4.0/17-20/01).

### Inclusion and exclusion criteria

The inclusion criteria were as follows: (i) patients aged ≥ 18 years; (ii) admitted within 24 h of ICH onset, and (iii) who provided informed consent. The exclusion criteria were as follows: (i) patients with severe aphasia, deafness, or unconsciousness, (ii) multiple organ dysfunction syndrome, (iii) secondary hemorrhage, (iv) bilateral hemorrhage, (v) intraventricular hemorrhage, or (vi) past dementia history.

### Sample size estimation

The ideal sample size for our study was estimated according to the sample size calculation formula of Geng et al. (2017), in which the prevalence of cognitive impairment was 12.7% (Nie et al., 2011) and the prevalence of PSCI was about 30% (Frances et al., 2016). Hence, the calculated sample size required for our study was 115.

### Cognition assessment

The clinical course of all participants was followed for a 6-month period after ICH to determine their cognitive function, which was assessed by a trained neuropsychologist. According to the standard of Experts Consensus on PSCI Management (Wang and Dong, 2021), patients with Montreal Cognition Assessment (MoCA) scores lower than 26 were classified in the PSCI group and the others were classified in the non-PSCI group. The MoCA scale has been validated as an effective and sensitive assessment method for the identification of mild cognition impairment (Pendlebury et al., 2012). The total MoCA score is 30 points and includes 5 points for visual-spatial and executive function, 3 points for naming, 6 points for attention, 3 points for language, 2 points for abstraction, 5 points for memory, and 6 points for orientation. For a patient with less than 12 years of education, an additional point is added to the test results to obtain a corrected MoCA score.

### Neurological function and neuroimaging evaluation

Neurological function and neuroimaging data were evaluated by an experienced neurologist. The National Institutes of Health Stroke Scale (NIHSS) was used to assess neurological function. Hematoma volume was calculated using the widely accepted ABC/2 formula. In addition, the severity of white matter hyperintensity (WMH) was rated *via* the fluid-attenuated inversion recovery (FLAIR) sequence from the magnetic resonance imaging (MRI) examination. WMH is defined as a focal lesion in the white matter with the corresponding hyperintensity on the FLAIR sequence. Based on the Fazekas scale, we assessed the presence and severity of deep and periventricular WMHs. Accordingly, WMHs were classified as none, minor, middle, or severe four grades (Pantoni et al., 2006).

### Statistical analysis

The Kolmogorov–Smirnov test and Levene’s test, respectively, were used to determine the normality and homogeneity of data. Then continuous variables were presented as mean ± standard deviation or median (interquartile range) and categorical variables were presented as frequencies (percentage). The Student’s *t*-test or Mann–Whitney *U*-test was performed to compare continuous variables between groups, while the Chi-square test was performed to compare categorical variables between groups. Multi-collinearity diagnostics (backward method) was used to identify and simplify the correlated independent variables between RBC indices. The risk factors for PSCI in ICH patients were screened using univariate and multivariate analyses. The backward likelihood ratio (***LR***) method was used to evaluate the strength according to the odds ratio (***OR***) and corresponding 95% confidence interval (***CI***), with the F probability of entry set at 0.05 and that of removal set at 0.10. All statistical analyses were performed using the SPSS software version 26.0 (IBM Corporation, New York, United States). ***P***-value < 0.05 was considered statistically significant.

The independent risk factors selected were used to establish a risk prediction model of PSCI. We formulated the nomogram based on the multivariate logistic regression using the R software version 3.6.3 (the R Foundation for Statistical Computing Platform). The prediction performance of the nomogram was measured using the following. The receiver operating characteristic (ROC) curve was employed to evaluate the model’s distinguishing ability between the PSCI and non-PSCI groups, the calibration curve was employed to evaluate the model’s accuracy, the decision curve analysis (DCA) was employed to evaluate the model’s clinical benefit and applicability, and the clinical impact curve (CIC) was employed to evaluate its predictive value.

## Results

### Cognitive function of the development and validation cohort

As shown in Figure 1, 259 patients of the development cohort who met the inclusion criteria were initially recruited in the stroke center of the Shanghai Tenth People’s Hospital from July 2017 to December 2018. Of these, 118 patients assessed without eligibility were excluded because of the exclusion criteria. After the 6-month follow-up after ICH for cognitive function assessment, a total of 123 patients were finally enrolled to develop the PSCI nomogram. Similarly, in the validation cohort, 137 patients met the inclusion criteria from July 2019 to February 2020, but 71 of them were excluded. Except for six patients lost to follow-up, 60 were eventually included to validate the PSCI nomogram. Among all the patients in these two cohorts, there were 69 (56.1%) and 38 (63.3%) identified as PSCI after 6-moth follow-up, respectively.

### Risk factors for post-stroke cognitive impairment in intracerebral hemorrhage patients in the development cohort

The baseline characteristics of the development cohort are showed in Table 1. Compared with the non-PSCI group patients, those identified as PSCI were older (69.96 vs. 63.11 years; ***P*** = 0.003), had lower education level (***P*** = 0.001) and larger hematoma volume (5.81 vs. 2.43 mL; ***P*** < 0.001), were more likely to have dominant-hemisphere hemorrhage (78.3% vs. 50.0%; ***P*** = 0.001) and lobar-hemorrhage (43.5% vs. 16.7%; ***P*** = 0.002), and had more severe WMH (***P*** = 0.008). Moreover, RBC indices such as total RBC count (4.30 × 10^12^ vs. 4.71 × 10^12^/L; ***P*** < 0.001), hemoglobin (HGB) (131.00 vs. 139.83 g/L; ***P*** = 0.008), mean corpuscular volume (MCV) (92.48 vs. 88.32 fL; ***P*** = 0.001), mean corpuscular hemoglobin concentration (MCHC) (331.26 vs. 335.83 g/L; ***P*** = 0.028), red cell distribution width (RDW) (13.38% vs. 13.01%; ***P*** = 0.034) were found to be significantly different between the PSCI and non-PSCI groups. Furthermore, multi-collinearity diagnostics recognized that HGB, MCV, and RDW were independent variables of RBC indices (Table 2). Lower low-density lipoprotein (LDL) levels were also significantly different between the PSCI and non-PSCI groups (2.21 vs. 2.61 mmol/L; ***P*** = 0.012).

**TABLE 1:** Characteristics comparison of PSCI and Non-PSCI groups in ICH patients.

Variables	All (*N* = 123)	PSCI (*N* = 69)	Non-PSCI (*N* = 54)	*P*-value
**Basic demographics**				
Age, years	66.95 ± 12.77	69.96 ± 12.03	63.11 ± 12.75	0.003*
Sex				0.610
Male	76 (61.8%)	44 (63.8%)	32 (59.3%)	
Female	47 (38.2%)	25 (36.2%)	22 (40.7%)	
Education level				0.001*
Illiteracy	3 (2.4%)	3 (4.3%)	0 (0.0%)	
Primary school	18 (14.6%)	6 (8.7%)	12 (22.2%)	
Middle school	36 (29.3%)	27 (39.1%)	9 (16.7%)	
High school	27 (22.0%)	18 (26.1%)	9 (16.7%)	
Higher	39 (31.7%)	15 (21.7%)	24 (44.4%)	
Excessive smoking	33 (26.8%)	18 (26.1%)	15 (27.8%)	0.834
Excessive drinking	29 (23.6%)	14 (20.3%)	15 (27.8%)	0.332
**Comorbid diseases**				
Hypertension	111 (90.2%)	63 (91.3%)	48 (88.9%)	0.654
Diabetes	45 (36.6%)	24 (34.8%)	21 (38.9%)	0.639
Coronary heart disease	33 (26.8%)	19 (27.5%)	14 (25.9%)	0.841
**Status on admission**				
NIHSS score	4 (2–6)	4 (2–7)	3.5 (2–5)	0.298
sBP, mmHg	160.59 ± 26.59	161.57 ± 26.95	159.33 ± 26.32	0.646
dBP, mmHg	89.59 ± 17.71	87.35 ± 17.92	92.44 ± 17.17	0.114
**Neuroimaging data**				
Hematoma volume, mL	3.71 (1.24–11.06)	5.81 (2.00–12.88)	2.43 (0.83–4.28)	<0.001*
Dominant-hemisphere hemorrhage	81 (65.9%)	54 (78.3%)	27 (50.0%)	0.001*
Lobar-hemorrhage	39 (31.7%)	30 (43.5%)	9 (16.7%)	0.002*
WMH				0.008*
None	30 (24.4%)	12 (17.4%)	18 (33.3%)	
Minor	24 (19.5%)	9 (13.0%)	15 (27.8%)	
Moderate	42 (34.1%)	30 (43.5%)	12 (22.2%)	
Severe	27 (22.0%)	18 (26.1%)	9 (16.7%)	
**Laboratory tests**				
CRP, mg/L	3.36 (3.17–5.50)	3.49 (3.13–6.43)	3.20 (3.25–5.45)	0.438
WBC, *10^9^ g/L	8.09 ± 2.68	8.36 ± 2.86	7.76 ± 2.40	0.221
RBC, *10^12^ g/L	4.48 ± 0.58	4.30 ± 0.58	4.71 ± 0.50	<0.001*
HGB, g/L	134.88 ± 18.00	131.00 ± 15.68	139.83 ± 19.64	0.008*
MCV, fL	90.65 ± 6.78	92.48 ± 6.92	88.32 ± 5.86	0.001*
MCH, pg	30.25 ± 2.60	30.66 ± 2.31	29.72 ± 2.85	0.045*
MCHC, g/L	333.27 ± 10.65	331.26 ± 6.94	335.83 ± 13.69	0.028*
RDW,%	13.22 ± 0.93	13.38 ± 0.81	13.01 ± 1.04	0.034*
PLT, *10^9^ g/L	193.41 ± 48.17	196.43 ± 53.58	189.56 ± 40.37	0.418
ALB, g/L	41.26 ± 3.18	41.00 ± 3.70	41.59 ± 2.34	0.281
ALT, U/L	16.87 ± 8.42	16.41 ± 8.05	17.44 ± 8.90	0.502
AST, U/L	19.46 ± 4.44	19.55 ± 4.69	19.33 ± 4.15	0.789
BUN, mmol/L	4.28 ± 1.37	4.22 ± 1.64	4.35 ± 0.92	0.560
CR, μmol/L	70.16 ± 20.41	67.59 ± 16.52	73.43 ± 24.28	0.116
UA, μmol/L	251.96 ± 76.55	246.65 ± 68.43	258.74 ± 86.02	0.387
TC, mmol/L	4.20 ± 0.90	4.06 ± 0.78	4.38 ± 1.02	0.062
TG, mmol/L	1.80 ± 0.94	1.69 ± 0.89	1.95 ± 0.99	0.118
LDL, mmol/L	2.39 ± 0.83	2.21 ± 0.71	2.61 ± 0.93	0.012*
FPG, mmol/L	6.03 ± 1.11	5.99 ± 1.17	6.08 ± 1.05	0.648
HbA1c,%	6.14 ± 0.78	6.08 ± 0.73	6.22 ± 0.85	0.334
HCY, μmol/L	13.19 ± 5.37	13.38 ± 5.78	12.95 ± 4.82	0.665

*P-value < 0.05.

**TABLE 2:** Multi-collinearity diagnostics of RBC indices.

	Beta	*t*	Collinearity statistics		*P*-value
			
			Tolerance	VIF	
RBC	–0.116	–0.851	0.331	3.020	0.397
HGB	–0.286	–3.413	0.878	1.139	0.001*
MCV	0.468	5.531	0.864	1.158	<0.001*
MCH	0.052	0.431	0.422	2.367	0.667
MCHC	–0.102	–1.219	0.870	1.149	0.225
RDW	0.264	3.080	0.841	1.189	0.003*
Constant	–	–3.358	–	–	0.001*

*P-value < 0.05.

In order to eliminate the non-linear influence between continuous variables and the outcome, we transferred above baseline characteristics into categorical variables before logistic regression analysis. Patients were divided in subgroups according to age (<50; 50–60; 60–70; 70–80; ≥ 80 years), hematoma volume (<5; 5–10; 10–20; ≥ 20 mL), HGB (<120; 120–160; ≥ 160 g/L for male and < 110; 110–150; ≥ 150 g/L for female), MCV (<80; 80–90; 90–100; ≥ 100 fL), RDW (<12.5%; ≥ 12.5%) and LDL (<1.8; 1.8–2.6; 2.6–3.4; ≥ 3.4 mmol/L). Table 3 shows the results of the univariate and multivariate logistic regression analyses. Seven potential predictors, including three RBC indices (RDW, HGB, and MCV), were screened by the multivariate analysis, while lobar-hemorrhage, WMH, and LDL were eliminated owing to their little significance. Age [***OR***: 1.779 (1.020–3.102); ***P*** = 0.042], education level [***OR***: 0.317 (0.136–0.737); ***P*** = 0.008], hematoma volume [***OR***: 3.550 (1.866–6.752); ***P*** < 0.001], dominant-hemisphere hemorrhage [***OR***: 10.296 (2.392–44.330); ***P*** = 0.002], HGB [***OR***: 0.166 (0.038–0.725); ***P*** = 0.017], MCV [***OR***: 10.204 (2.369–43.956); ***P*** = 0.002], RDW [***OR***:18.055 (3.784–86.151); ***P*** < 0.001] were determined as independent risk factors for PSCI. Table 4 shows the performance of these seven risk factors, in terms of sensitivity, specificity, and area under the curve (AUC) to evaluate their diagnosis utility.

**TABLE 3:** Univariate and multivariate logistic regression analysis to predict PSCI in ICH patients.

Categorical variables	Univariate analysis		Multivariate analysis	
		
	OR (95% CI)	*P*-value	OR (95% CI)	*P*-value
Age	1.842 (1.329–2.551)	<0.001	1.779 (1.020–3.102)	0.042*
Education level	0.783 (0.569–1.079)	0.135	0.317 (0.136–0.737)	0.008*
Hematoma volume	2.097 (1.378–3.191)	0.001	3.550 (1.866–6.752)	<0.001*
Dominant-hemisphere hemorrhage	3.600 (1.647–7.870)	0.001	10.296 (2.392–44.330)	0.002*
Lobar-hemorrhage	3.846 (1.628–9.085)	0.002	–	–
WMH	1.639 (1.158–2.319)	0.005	–	–
HGB	0.409 (0.208–0.801)	0.009	0.166 (0.038–0.725)	0.017*
MCV	2.737 (1.524–4.914)	0.001	10.204 (2.369–43.956)	0.002*
RDW	5.333 (2.206–12.891)	<0.001	18.055 (3.784–86.151)	<0.001*
LDL	0.702 (0.486–1.103)	0.059	–	–

*P-value < 0.05.

**TABLE 4:** Performance of each prognostic factor of PSCI in ICH patients.

	Cut-off value	Sensitivity	Specificity	AUC	95% CI	*P*-value
Age, years	68.5	0.609	0.741	0.665	0.567–0.763	0.002*
Education level	High school-Higher	0.444	0.783	0.579	0.473–0.684	0.136
Hematoma volume, mL	4.47	0.594	0.778	0.688	0.595–0.781	<0.001*
Dominant-hemisphere hemorrhage	Yes-No	0.783	0.500	0.641	0.541–0.741	0.007*
HGB, g/L	145.0	0.444	0.870	0.619	0.516–0.722	0.024*
MCV, fL	93.55	0.478	0.944	0.740	0.653–0.826	<0.001*
RDW,%	12.45	0.870	0.444	0.611	0.507–0.715	0.035*

*P-value < 0.05.

### Construction of a nomogram incorporating red blood cell indices to predict post-stroke cognitive impairment in intracerebral hemorrhage patients

**FIGURE 2:**
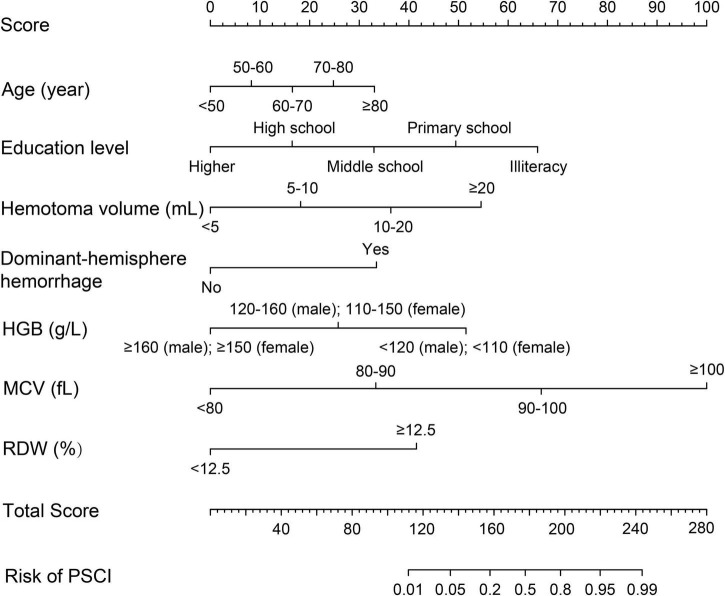
Prognostic nomogram for PSCI in ICH patients. The value of an individual patient is located on each variable axis, and a line is drawn upward to the “Score” axis to determine the corresponding score received for each variable value. The total score is calculated by adding each score of the seven variables included in the nomogram, and a line is drawn from the “Total Score” axis downward to the “Risk of PSCI” axis to determine the likelihood of PSCI.

We combined risk factors to construct three different prediction models in relation to PSCI. Model 1 incorporated age, education level, hematoma volume, and dominant-hemisphere hemorrhage, Model 2 incorporated RBC indices (HGB, MCV, and RDW), and Model 3 was the combination of Model 1 and Model 2. As demonstrated in Table 5, compared with Model 1 and Model 2, Model 3 had the largest area under the ROC curve to identify PSCI, with AUC 0.940 (0.893–0.987). Therefore, as the combined Model 3 had the best predictive performance, we built a nomogram incorporating RBC indices based on Model 3. A summary of the point value of each factor used to calculate the total score is presented in Figure 2. The length of each variable line illustrates its relative importance for the risk of PSCI. With each factor value given a score on the “Score” scale, we calculate the total score by adding each of the seven factors in the nomogram. By projecting the total score to the lower “Risk of PSCI” scale, we are able to estimate the probability of PSCI.

**TABLE 5:** Comparison of different models to predict PSCI in ICH patients.

	SE	AUC	95% CI	*P*-value
Model 1	0.038	0.853	0.779–0.927	<0.001*
Model 2	0.038	0.812	0.738–0.885	<0.001*
Model 3	0.024	0.940	0.893–0.987	<0.001*

Model 1: Age + Education level + Hematoma volume + Dominant-hemisphere hemorrhage; Model 2: RBC indices (HGB + MCV + RDW); Model 3: Model 1 + Model 2.

*P-value < 0.05.

### Performance evaluation of the post-stroke cognitive impairment nomogram incorporating red blood cell indices

**FIGURE 3:**
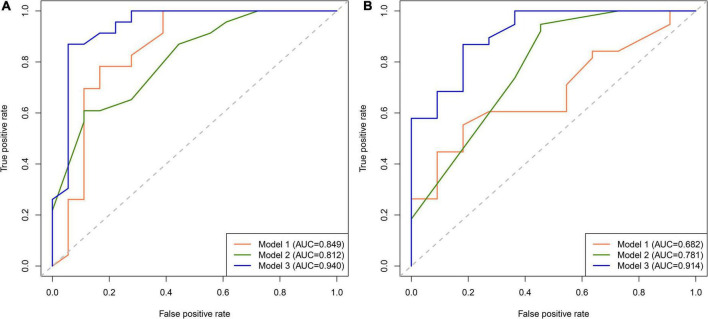
Receiver operating characteristic (ROC) curve for PSCI nomogram. **(A)** Development cohort; **(B)** validation cohort.

**FIGURE 4:**
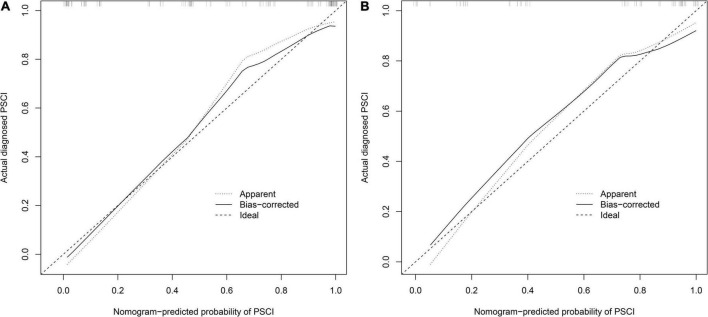
Calibration curve for PSCI nomogram. **(A)** Development cohort; **(B)** validation cohort. The nomogram-predicted probability of PSCI is plotted on the x-axis and that of actual PSCI is plotted on the y-axis.

**FIGURE 5:**
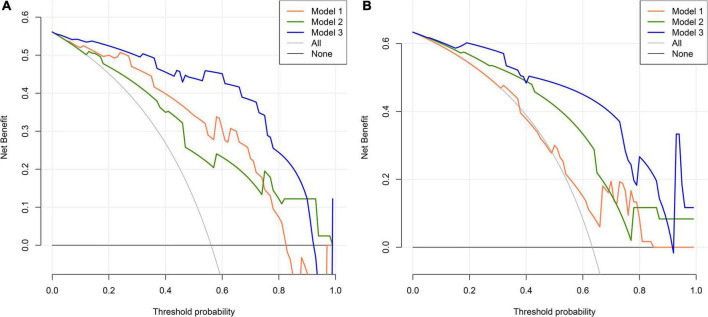
Decision curve analysis (DCA) for PSCI nomogram. **(A)** Development cohort; **(B)** validation cohort. The abscissa represents the threshold probability and the y-axis represents the net benefit.

**FIGURE 6:**
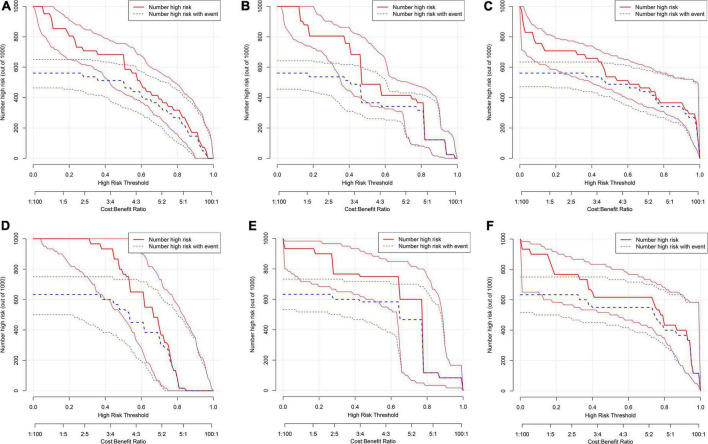
Clinical impact curve (CIC) for PSCI nomogram. **(A–C)** Development cohort: model 1 **(A)**, model 2, **(B)** model 3 **(C)**; **(D–F)** validation cohort: model 1 **(D)**, model 2 **(E)**, model 3 **(F)**. The red curve (number of high-risk individuals) indicates the number of people who are classified as positive (high risk) by the model at each threshold probability; the blue curve (number of high-risk individuals with outcome) is the number of true positives at each threshold probability.

We first compared the three models using ROC analysis. The AUC indicating the predictive capacity of the prediction model was 0.849 (Model 1), 0.812 (Model 2), and 0.940 (Model 3) in the development cohort (Figure 3A) and 0.682 (Model 1), 0.781 (Model 2), and 0.914 (Model 3) in the validation cohort (Figure 3B). Altogether, this suggests that Model 3 was reasonably the most accurate for good discrimination ability between the PSCI and non-PSCI groups. Alternatively, the calibration curve of Model 3 revealed an adequate fit of the nomogram predicting the actual risk of PSCI with the ideal curve, indicating its good predictive performance (Figures 4A,B). DCA revealed that across the range of threshold probability, nomogram-assisted decisions to assess PSCI provided a significant net benefit in clinical decision-making. Additionally, Model 3 showed more applicability than Model 1 and Model 2 in both the development and validation cohort (Figures 5A,B). On the basis of the DCA, a CIC was plotted using the cost-benefit ratio to visualize the number of patients at high risk for PCSI and the proportion of those who were true positives. The number of high-risk patients in Model 3 was closer to the actual number of true-positive patients than in Model 1 and Model 2 in both development and validation cohorts (Figures 6A–F).

## Discussion

In this study, we conducted a comprehensive analysis to determine the potential relationship between RBC indices and PCSI in ICH patients. We established a model incorporating RBC indices to accurately predict PSCI risk and facilitate clinical decision-making by integrating multivariable effects.

Previous studies have proved the significance of RBC indices in predicting mortality, clinical outcomes, and functional rehabilitation after stroke. Post-stroke outcomes have been associated with RBC indices (Winchester et al., 2018; Drozdowska et al., 2020; Gong et al., 2020). For instance, RDW is closely related to the outcome of carotid atherosclerosis and ischemic stroke (Chugh et al., 2015; Li et al., 2017; Turcato et al., 2017). It is also reported that RDW is an independent predictor of 30-day mortality in ICH patients (Pinho et al., 2021). Altintas et al. (2017) showed elevated RDW levels to be significantly positively correlated with hematoma growth in a retrospective study based on 60 patients with cerebral parenchymal hemorrhage. Simultaneous, Hong et al. (2018) also conducted a prospective cohort study in 364 patients with aneurysmal subarachnoid hemorrhage and found that elevated RDW levels were predictive of poor prognosis. Moreover, clinical investigations suggested that the use of RDW in conjunction with MCV showed better predictive performances in chronic disease prognosis compared with either RDW or MCV alone (Kor et al., 2018). Decreased HGB level has been shown to be a risk factor for poor prognosis after hemorrhagic stroke in recent studies (Diedler et al., 2010; Gong et al., 2020). In addition, there is increasing evidence that RBC indices are also related to cognitive impairment. Anemia or low RBC count has been associated with mild cognitive impairment risk (Dlugaj et al., 2016). Corroborating the findings of these studies, our findings suggest that RDW, MCV, and HGB are independent risk factors for PSCI.

This study is our second attempt to evaluate the relationship between RBC indices and PSCI occurrence, and the results are in line with our previous work (Gong et al., 2020). After the adjustment of covariates, lower HGB [***OR***: 0.166 (0.038–0.725); ***P*** = 0.017], larger MCV [***OR***: 10.204 (2.369–43.956); ***P*** = 0.002] and wider RDW [***OR***:18.055 (3.784–86.151); ***P*** < 0.001] remained associated with PSCI in ICH patients. RBC indices, including HGB, MCV, and RDW, may share a series of cognitive impairment mechanisms, such as hypoxia, iron dyshomeostasis, and folic acid and vitamin deficiencies. Several underlying mechanisms may explain as follows.

HGB is the carrier of oxygen and participates in oxygen transport. HGB abnormalities will deprive oxygen delivery to the brain and thus attenuate neuronal energy metabolism (Weiss et al., 2022). Deficient oxygen transport may trigger hypoperfusion, cerebral blood flow changes, and disorder of cerebral auto-regulation, which may further lead to cerebral degenerative abnormalities, such as cognitive dysfunction (Wan et al., 2020). In addition, lower HGB tends to be related to iron deficiency anemia, which may facilitate cognitive decline by affecting the synthesis of neurotransmitters and catecholamine metabolism (Petranovic et al., 2008; Yavuz et al., 2012).

MCV represents the average size or volume of circulating erythrocytes. Larger RBCs may have difficulty manipulating their shape to fit through capillaries and transport oxygen and nutrients, especially in the brain regions connected with memory performance in older adults (Gamaldo et al., 2013). Moreover, the abnormal status of cell volume augments RBC fragility, which accelerates cell rupture and triggers iron dyshomeostasis after ICH onset (Duce et al., 2010; Gong et al., 2019). The iron overload promotes reactive oxygen species generation and amyloid formation (Cheignon et al., 2018), and further affects cognitive function.

RDW reflects the heterogeneity of circulating erythrocyte volume (Patel et al., 2013; Balci et al., 2016). An elevated RDW is related to impaired erythropoiesis or erythrocyte degradation (Öztürk et al., 2013) and indicates a more mixed population of RBC volumes (Patel et al., 2010). Because of decreased deformability and increased fragility of erythrocytes, high RDW causes insufficient oxygen supply and leads to hypoxia (Patel et al., 2013). An elevated RDW is often related to anemia due to nutrient deficiencies, such as iron, vitamin B12, and folic acid (Buttarello, 2016). Vitamin B12 and folic acid deficiencies result in hyperhomocysteinemia, which is associated with cerebrovascular diseases and dementia (Baroni et al., 2019).

Nomograms are designed based on the comprehensive generalization of risk factors to intuitively and conveniently predict clinical outcomes (Balachandran et al., 2015). In this study, we assessed the association between RBC indices and PSCI and constructed a nomogram for predicting the occurrence of PSCI. Additionally, we evaluated the performance of the constructed nomogram based on RBC indices in predicting PSCI occurrence and found it to be effective. Therefore, we postulate that the RBC indices, measured as part of the most common and convenient commercial laboratory examinations could efficiently predict cognitive function after ICH. We suggest that the nomogram constructed in this study by combining notable risk factors in PSCI prediction may aid clinicians in predicting patients’ cognitive outcomes and help clinicians make reasonable treatment decisions and adjust individualized therapy. However, there were some limitations to our work. First, patients with severe hemiplegia and aphasia after ICH could not be evaluated by comprehensive neuropsychological examination and should be assessed to avoid bias. Second, this is a single-center study and the findings may not represent that of other regions. Therefore, our findings must be validated in a prospective international multicenter study to increase the credibility.

## Conclusion

Our study revealed that RBC indices are significantly independent predictors of PSCI. A nomogram incorporating RBC indices (HGB, MCV, RDW) and other relevant risk factors (age, education level, hematoma volume, dominant-hemisphere hemorrhage) can aid the early and accurate prediction of the occurrence of PSCI in the ICH population. The nomogram established in this study can be used as a reasonable and reliable graphic tool to help clinicians identify high cognition impairment-risk patients and adjust individualized therapy for ICH patients.

## Data availability statement

The original contributions presented in this study are included in the article/supplementary material, further inquiries can be directed to the corresponding author.

## Ethics statement

The studies involving human participants were reviewed and approved by the Ethics Committee of Shanghai Tenth People’s Hospital. The patients/participants provided their written informed consent to participate in this study.

## Author contributions

XL and MF made the conception and designed the experiment. LG provided the administrative support. YG and FW collected and assembled the raw data. YG analyzed the data and wrote the manuscript. XL funded the experiment. All authors approved the final manuscript and agreed to be accountable for the content of the work.

## Abbreviations

PSCI: post-stroke cognitive impairment
ICH: intracerebral hemorrhage
NIHSS: National Institute of Health stroke scale
Sbp: systolic blood pressure
dBP: diastolic blood pressure
WMH: white matter hyperintensities
CRP: C-reactive protein
WBC: white blood cell
RBC: red blood cell
HGB: hemoglobin
MCV: mean corpuscular volume
MCH: mean corpuscular hemoglobin
MCHC: mean corpuscular hemoglobin concentration
HCT: hematocrit
RDW: red cell distribution width
PLT: platelet
PCT: plateletcrit
PDW: platelet distribution width
ALB: albumin
ALT: alanine aminotransferase
AST: aspartate aminotransferase
BUN: blood urea nitrogen
CR: creatinine
eGFR: estimated glomerular filtration rate
UA: uric acid
TC: total cholesterol
TG: triglycerides
HDL: high density lipoprotein
LDL: low density lipoprotein
FPG: fasting plasma glucose
HbA1c: hemoglobin A1c
HCY: homocysteine
AUC: area under the curve
ROC: receiver operating characteristic
DCA: decision curve analysis
CIC: clinical impact curve.

## References

[B1] Altintas O. Duruyen H. Baran G. Baran O. Katar S. Antar V. (2017). The relationship of hematoma growth to red blood cell distribution width in patients with hypertensive intracerebral hemorrhage. *Turk Neurosurg.* 27 368–373. 27593780 10.5137/1019-5149.JTN.16136-15.1

[B2] Balachandran V. P. Gonen M. Smith J. J. Dematteo R. P. (2015). Nomograms in oncology: more than meets the eye. *Lancet Oncol.* 16 e173–e180. 10.1016/S1470-2045(14)71116-7 25846097 PMC4465353

[B3] Balci Y. I. Akpinar F. O. Polat A. Uzun U. Ergin A. (2016). Evaluation of reticulocyte parameters in iron deficiency, vitamin B12 deficiency and mixed anemia. *Clin. Lab.* 62 343–347. 10.7754/Clin.Lab.2015.150616 27156322

[B4] Baroni L. Bonetto C. Rizzo G. Bertola C. Caberlotto L. Bazzerla G. (2019). Association between cognitive impairment and vitamin B12, folate, and homocysteine status in elderly adults: a retrospective study. *J. Alzheimers Dis.* 70 443–453. 10.3233/JAD-190249 31177227

[B5] Buttarello M. (2016). Laboratory diagnosis of anemia: are the old and new red cell parameters useful in classification and treatment, how? *Int. J. Lab. Hematol.* 38 123–132. 10.1111/ijlh.12500 27195903

[B6] Cheignon C. Tomas M. Bonnefont-Rousselot D. Faller P. Hureau C. Collin F. (2018). Oxidative stress and the amyloid beta peptide in Alzheimer’s disease. *Redox. Biol.* 14 450–464. 10.1016/j.redox.2017.10.014 29080524 PMC5680523

[B7] Chugh C. Nyirjesy S. C. Nawalinski K. P. Sandsmark D. K. Frangos S. Maloney-Wilensky E. (2015). Red blood cell distribution width is associated with poor clinical outcome after subarachnoid hemorrhage: a pilot study. *Neurocrit. Care* 23 217–224. 10.1007/s12028-015-0117-x 25672971

[B8] Diedler J. Sykora M. Hahn P. Heerlein K. Scholzke M. N. Kellert L. (2010). Low hemoglobin is associated with poor functional outcome after non-traumatic, supratentorial intracerebral hemorrhage. *Crit. Care* 14:R63. 10.1186/cc8961 20398266 PMC2887185

[B9] Dlugaj M. Winkler A. Weimar C. Dürig J. Broecker-Preuss M. Dragano N. (2016). Anemia and mild cognitive impairment in the german general population. *J. Alzheimers Dis.* 49 1031–1042. 10.3233/JAD-150434 26599053

[B10] Dong Y. Ding M. Cui M. Fang M. Gong L. Xu Z. (2021). Development and validation of a clinical model (DREAM-LDL) for post-stroke cognitive impairment at 6 months. *Aging* 13 21628–21641. 10.18632/aging.203507 34506303 PMC8457606

[B11] Drozdowska B. A. Elliott E. Taylor-Rowan M. Shaw R. C. Cuthbertson G. Langhorne P. (2020). Cardiovascular risk factors indirectly affect acute post-stroke cognition through stroke severity and prior cognitive impairment: a moderated mediation analysis. *Alzheimers Res. Ther.* 12:85. 10.1186/s13195-020-00653-y 32678028 PMC7367370

[B12] Duce J. A. Tsatsanis A. Cater M. A. James S. A. Robb E. Wikhe K. (2010). Iron-export ferroxidase activity of beta-amyloid precursor protein is inhibited by zinc in Alzheimer’s disease. *Cell* 142 857–867. 10.1016/j.cell.2010.08.014 20817278 PMC2943017

[B13] Frances A. Sandra O. Lucy U. (2016). Vascular cognitive impairment, a cardiovascular complication. *World J. Psychiatry* 6 199–207. 10.5498/wjp.v6.i2.199 27354961 PMC4919258

[B14] Gamaldo A. A. Ferrucci L. Rifkind J. Longo D. L. Zonderman A. B. (2013). Relationship between mean corpuscular volume and cognitive performance in older adults. *J. Am. Geriatr. Soc.* 61 84–89. 10.1111/jgs.12066 23301873 PMC3555566

[B15] Geng S. Liu N. Meng P. Ji N. Sun Y. Xu Y. (2017). Midterm blood pressure variability is associated with poststroke cognitive impairment: a prospective cohort study. *Front. Neurol.* 8:365. 10.3389/fneur.2017.00365 28804475 PMC5532726

[B16] Gilev K. V. Yastrebova E. S. Strokotov D. I. Yurkin M. A. Karmadonova N. A. Chernyshev A. V. (2017). Advanced consumable-free morphological analysis of intact red blood cells by a compact scanning flow cytometer. *Cytometry A* 91 867–873. 10.1002/cyto.a.23141 28544427

[B17] Gong L. Gu Y. Yu Q. Wang H. Zhu X. Dong Q. (2020). Prognostic factors for cognitive recovery beyond early Poststroke Cognitive Impairment (PSCI): a prospective cohort study of spontaneous intracerebral hemorrhage. *Front. Neurol.* 11:278. 10.3389/fneur.2020.00278 32411073 PMC7198781

[B18] Gong L. Tian X. Zhou J. Dong Q. Tan Y. Lu Y. (2019). Iron dyshomeostasis induces binding of APP to BACE1 for amyloid pathology, and impairs APP/Fpn1 complex in microglia: implication in pathogenesis of cerebral microbleeds. *Cell Transplant.* 28 1009–1017. 10.1177/0963689719831707 30776900 PMC6728710

[B19] Gong L. Wang H. Zhu X. Dong Q. Yu Q. Mao B. (2021). Nomogram to predict cognitive dysfunction after a minor ischemic stroke in hospitalized-population. *Front. Aging Neurosci.* 13:637363. 10.3389/fnagi.2021.637363 33967738 PMC8098660

[B20] Hachinski V. Iadecola C. Petersen R. C. Breteler M. M. Nyenhuis D. L. Black S. E. (2006). National institute of neurological disorders and stroke-canadian stroke network vascular cognitive impairment harmonization standards. *Stroke* 37 2220–2241. 10.1161/01.STR.0000237236.88823.47 16917086

[B21] Hong D. Y. Kim S. Y. Kim J. Y. Kim J. W. (2018). Red blood cell distribution width is an independent predictor of mortality in patients with aneurysmal subarachnoid hemorrhage. *Clin. Neurol. Neurosurg.* 172 82–86. 10.1016/j.clineuro.2018.06.044 29986200

[B22] Keep R. F. Hua Y. Xi G. (2012). Intracerebral haemorrhage: mechanisms of injury and therapeutic targets. *Lancet Neurol.* 11 720–731. 10.1016/S1474-4422(12)70104-7 22698888 PMC3884550

[B23] Kor C. T. Hsieh Y. P. Chang C. C. Chiu P. F. (2018). The prognostic value of interaction between mean corpuscular volume and red cell distribution width in mortality in chronic kidney disease. *Sci. Rep.* 8:11870. 10.1038/s41598-018-19881-2 30089848 PMC6082905

[B24] Li N. Zhou H. Tang Q. (2017). Red blood cell distribution width: a novel predictive indicator for cardiovascular and cerebrovascular diseases. *Dis. Mark.* 2017:7089493. 10.1155/2017/7089493 29038615 PMC5606102

[B25] Lv T. Zhao B. Hu Q. Zhang X. (2021). The glymphatic system: a novel therapeutic target for stroke treatment. *Front. Aging Neurosci.* 13:689098. 10.3389/fnagi.2021.689098 34305569 PMC8297504

[B26] Nie H. Xu Y. Liu B. Zhang Y. Lei T. Hui X. (2011). The prevalence of mild cognitive impairment about elderly population in China: a meta-analysis. *Int. J. Geriatr. Psychiatry* 26 558–563. 10.1002/gps.2579 20878675

[B27] Öztürk Z. A. Ünal A. Yiğiter R. Yesil Y. Kuyumcu M. E. Neyal M. (2013). Is increased red cell distribution width (RDW) indicating the inflammation in Alzheimer’s disease (AD)? *Arch. Gerontol. Geriatr.* 56 50–54. 10.1016/j.archger.2012.10.002 23103090

[B28] Pantoni L. Poggesi A. Basile A. M. Pracucci G. Barkhof F. Chabriat H. (2006). Leukoaraiosis predicts hidden global functioning impairment in nondisabled older people: the LADIS (Leukoaraiosis and Disability in the Elderly) Study. *J. Am. Geriatr. Soc.* 54 1095–1101. 10.1111/j.1532-5415.2006.00798.x 16866681

[B29] Pasi M. Sugita L. Xiong L. Charidimou A. Boulouis G. Pongpitakmetha T. (2021). Association of cerebral small vessel disease and cognitive decline after intracerebral hemorrhage. *Neurology* 96 e182–e192. 10.1212/WNL.0000000000011050 33067403 PMC7905779

[B30] Patel K. V. Mohanty J. G. Kanapuru B. Hesdorffer C. Ershler W. B. Rifkind J. M. (2013). Association of the red cell distribution width with red blood cell deformability. *Adv. Exp. Med. Biol.* 765 211–216. 10.1007/978-1-4614-4989-8_29 22879035 PMC5939938

[B31] Patel K. V. Semba R. D. Ferrucci L. Newman A. B. Fried L. P. Wallace R. B. (2010). Red cell distribution width and mortality in older adults: a meta-analysis. *J. Gerontol. A Biol. Sci. Med. Sci.* 65 258–265. 10.1093/gerona/glp163 19880817 PMC2822283

[B32] Pendlebury S. T. Mariz J. Bull L. Mehta Z. Rothwell P. M. (2012). MoCA, ACE-R, and MMSE versus the national institute of neurological disorders and stroke-canadian stroke network vascular cognitive impairment harmonization standards neuropsychological battery after TIA and stroke. *Stroke* 43 464–469. 10.1161/STROKEAHA.111.633586 22156700 PMC5390857

[B33] Pendlebury S. T. Rothwell P. M. (2009). Prevalence, incidence, and factors associated with pre-stroke and post-stroke dementia: a systematic review and meta-analysis. *Lancet Neurol.* 8 1006–1018. 10.1016/S1474-4422(09)70236-4 19782001

[B34] Petranovic D. Batinac T. Petranovic D. Ruzic A. Ruzic T. (2008). Iron deficiency anaemia influences cognitive functions. *Med. Hypotheses* 70 70–72. 10.1016/j.mehy.2007.04.029 17574345

[B35] Pinho J. Silva L. Quintas-Neves M. Marques L. Amorim J. M. Reich A. (2021). Red cell distribution width is associated with 30-day mortality in patients with spontaneous intracerebral hemorrhage. *Neurocrit. Care* 34 825–832. 10.1007/s12028-020-01103-1 32959199 PMC8179905

[B36] Roh D. J. Albers D. J. Magid-Bernstein J. Doyle K. Hod E. Eisenberger A. (2019). Low hemoglobin and hematoma expansion after intracerebral hemorrhage. *Neurology* 93 e372–e380. 10.1212/WNL.0000000000007820 31209179 PMC6669932

[B37] Sun J. H. Tan L. Yu J. T. (2014). Post-stroke cognitive impairment: epidemiology, mechanisms and management. *Ann. Transl. Med.* 2:80. 10.3978/j.issn.2305-5839.2014.08.05 PMC4200648 25333055

[B38] Turcato G. Cappellari M. Follador L. Dilda A. Bonora A. Zannoni M. (2017). Red blood cell distribution width is an independent predictor of outcome in patients undergoing thrombolysis for ischemic stroke. *Semin. Thromb. Hemost.* 43 30–35. 10.1055/s-0036-1592165 27813042

[B39] Wan J. Luo P. Du X. Yan H. (2020). Preoperative red cell distribution width predicts postoperative cognitive dysfunction after coronary artery bypass grafting. *Biosci. Rep.* 40:BSR20194448. 10.1042/BSR20194448 32271371 PMC7178207

[B40] Wang K. Dong Q. (2021). Experts consensus on post-stroke cognitive impairment management 2021. *Chin. J. Stroke* 16, 376–389.

[B41] Weiss A. Beloosesky Y. Gingold-Belfer R. Leibovici-Weissman Y. Levy Y. Mulla F. (2022). Association of anemia with dementia and cognitive decline among community-dwelling elderly. *Gerontology* [Epub ahead of print]. 10.1159/000522500 35316810 PMC9808713

[B42] Winchester L. M. Powell J. Lovestone S. Nevado-Holgado A. J. (2018). Red blood cell indices and anaemia as causative factors for cognitive function deficits and for Alzheimer’s disease. *Genome Med.* 10 51. 10.1186/s13073-018-0556-z 29954452 PMC6022699

[B43] YavuzB. B.CankurtaranM.HaznedarogluI. C.HalilM.UlgerZ.AltunB. (2012). Iron deficiency can cause cognitive impairment in geriatric patients. *J. Nutr. Health Aging* 16 220–224. 10.1007/s12603-011-0351-7 22456776

[B44] Yuan X. Zhang L. Sui R. Wang Z. (2021). A risk prediction model of post-stroke cognitive impairment based on magnetic resonance spectroscopy imaging. *Neurol. Res.* 43 642–652. 10.1080/01616412.2021.1908659 33784942

[B45] Zhang X. Bi X. (2020). Post-stroke cognitive impairment: a review focusing on molecular biomarkers. *J. Mol. Neurosci.* 70 1244–1254. 10.1007/s12031-020-01533-8 32219663

[B46] Zheng F. Yan L. Zhong B. Yang Z. Xie W. (2019). Progression of cognitive decline before and after incident stroke. *Neurology* 93 e20–e28. 10.1212/WNL.0000000000007716 31127071

[B47] Zhou S. Chen J. Cheng L. Fan K. Xu M. Ren W. (2021). Age-dependent association between elevated homocysteine and cognitive impairment in a post-stroke population: a prospective study. *Front. Nutr.* 8:691837. 10.3389/fnut.2021.736283 34277686 PMC8284187

